# Structural and mechanistic characterization of heparin interactions with tau fibrils

**DOI:** 10.1016/j.jbc.2026.111153

**Published:** 2026-01-12

**Authors:** Fiona Mon, John E. Straub

**Affiliations:** Department of Chemistry, Boston University, Boston, Massachusetts, USA

**Keywords:** Tau protein, Alzheimer's disease, amyloid, fibril, heparin, molecular dynamics, Brownian dynamics

## Abstract

Soluble microtubule-associated tau protein can misfold and assemble into stable, insoluble amyloid fibrils. The accumulation of tau amyloid fibrils within neurons is a primary feature in the progression of neurodegenerative diseases, including Alzheimer’s disease. Tau fibrils have been observed to colocalize with glycosaminoglycans, such as heparan sulfate (HS), *in vivo*. Heparin is a highly sulfated analog of HS that has been commonly used *in vitro* to accelerate tau aggregation. The binding of heparin to tau fibrils inhibits fibril uptake by neighboring cells, whereas HS on the cell surface modulates this uptake. Understanding the molecular interactions of heparin and HS with tau fibrils is important in developing therapeutic targets that can slow the progression of neurodegeneration. In this multiscale computational study, we employ a combination of Brownian dynamics and molecular dynamics to simulate heparin binding to two tau fibril polymorphic structures. Our simulations lead to the *de novo* prediction of heparin binding to basic residue ladders organized along the tau fibril axis. The mechanism of binding is facilitated by long-range electrostatic steering of the polyanionic heparin to the tau fibril surface, followed by the refinement of favorable short-range heparin–tau interactions. The identified binding sites are located in regions of excess densities in cryo-EM maps of the tau fibrils, providing support for the computational predictions. Our findings provide a structural and mechanistic framework for a better understanding of fibril–glycan interactions and how they influence the overall mechanism of tau fibril propagation.

Amyloid fibrils are prevalent in the most common neurodegenerative diseases, such as Alzheimer’s disease (AD) and Parkinson’s disease, in which misfolding of soluble proteins results in stable, insoluble amyloid structures that ultimately lead to neuronal death (1, 2, 3). Tau is a functionally diverse, intrinsically disordered protein found primarily in neurons that plays an important role in microtubule stabilization and regulation of microtubule dynamics (3, 4). Six tau isoforms are present in an adult human brain that vary in sequence length, ranging from 352 to 441 amino acids, depending on the number of N-terminal inserts (N1 and N2) and the presence of a repeat motif R2 in the microtubule-binding domain (Fig. 1*A*) (5). Abnormal accumulation of tau fibrils in the brain is a prominent feature of neurodegenerative diseases referred to as tauopathies. AD is an example of a tauopathy and involves two pathological hallmarks: amyloid-β plaques and neurofibrillary tangles consisting of tau paired helical filaments (PHF polymorph) and straight filaments (SF polymorph) (6, 7).

**Figure 1:**
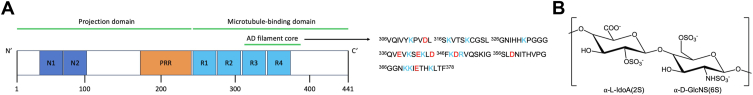
**Overview of full-length tau and heparin disaccharide unit.***A,* domain arrangement of full-length tau. Sequence of fibril core comprising residues 306 to 378 with basic residues highlighted in *blue* and acidic residues highlighted in *red*. *B,* molecular structure of a heparin disaccharide unit from a heparin chain length of four disaccharide units used in simulations.

*In vitro* studies have used various biomolecules to accelerate the formation of tau filaments, since tau is highly soluble and does not aggregate in the absence of truncation, cofactors, or seeding (8, 9, 10, 11, 12). PHFs alone are insufficient to template additional monomers, underscoring the requirement for other biomolecular components to establish the pathological properties of tau fibrils (13). However, it has been difficult to determine the significance of these biomolecules *in vivo* from the formation of tau filaments *in vitro*, as these *in vitro* models structurally differ from the tau amyloid fibrils derived from AD brain. A previous study has shown the importance of polyanionic cofactors in stabilizing mature tau fibrils, where fibril depolymerization was observed when cofactors were degraded (14). Tau is an intracellular protein, and typical polyanionic cofactors used *in vitro*, such as heparin and heparan sulfate proteoglycans (HSPGs), are found in the extracellular matrix. HSPGs have been identified to codeposit with tau fibrils extracted from AD brain tissue, suggesting that HSPGs must interact with tau at some point in the amyloid pathway (15, 16, 17, 18, 19). The biological relevance of the interaction of tau and heparin–HSPGs *in vivo* can only occur if tau is also present in the extracellular space, which has been previously proposed to elucidate the mechanism of the spread of tau aggregates in neurons (20). Experimental evidence suggests that the mechanism of propagation of tau aggregates is prion like, where intracellular pathological tau aggregates that are capable of seeding are released into the extracellular space, and the uptake of these tau aggregates in neighboring cells seeds intracellular fibrillization (21, 22, 23). The entry of tau aggregates into adjacent cells is mediated by HSPGs on the cell surface (21, 22, 23). Furthermore, heparin has been found to competitively inhibit tau binding to HSPGs, preventing tau fibril uptake (22). These findings suggest the importance of understanding the molecular interactions of heparin- and HS-tau fibril for potential therapeutic targets in tauopathies.

HSPGs consist of heparan sulfate (HS) glycosaminoglycan (GAG) chains covalently linked to a core protein on the cell surface. HS is a linear, polyanionic GAG that contains repeating disaccharide units composed of uronic acid (glucuronic acid or iduronic acid) sulfated at the 2-O position and glucosamine sulfated at the 3-O, 6-O, and N positions (Fig. 1*B*). Sulfation at the 3-O position of glucosamine has been reported to be relatively rare compared with other sulfated positions (24). Specific sulfation patterns affect the binding of HS to tau. Heparin mimics the highly sulfated regions of HS in which the predominant uronic acid component is l-iduronic acid, whereas d-glucuronic acid predominates in HS. In heparin, the 6-O position in the glucosamine is predominantly sulfated (25). Because of the age-associated increase in HS sulfation, heparin is often employed as the *in vitro* analog (26, 27).

Amyloid fibrils are comprised of parallel, in-register β-sheets along the fibril axis, which is essential to form periodic arrays of identical residues with an interstrand spacing of approximately 4.8 Å (28, 29). The periodic arrangement of heparin–HS has been proposed to accelerate amyloid fibril formation through a scaffolding-based mechanism to relieve the electrostatic repulsion of positively charged identical side chains along the fibril axis (30, 31, 32). Heparin rapidly induces heterogeneous, highly flexible oligomers, which are increasingly recognized as central mediators of toxicity in AD pathogenesis (33, 34, 35, 36). For PHF fibrils, it has been observed that heparin is an integral component of the fibril core and that free heparin does not interact with intrinsically disordered regions of the fibril (32). Multiple heparin binding sites within the core of tau AD fibrils have been proposed on tau monomers (32, 37).

Cryo-EM has been used to resolve many atomic structures of amyloid filaments and reveal the polymorphism of various amyloid fibrils containing distinct protofilament folds characteristic of different amyloid-forming diseases (38, 39, 40, 41, 42, 43). Regions of additional densities in cryo-EM maps may indicate post-translational modifications (44, 45) or unidentified elements that colocalize with amyloid fibrils (40, 46, 47, 48, 49). The additional densities surrounding the fibril core of α-synuclein have been shown to be reminiscent of lipids (47). Furthermore, the additional density within the α-synuclein fibril core of the protofilaments has been modeled with heparin (50) and polyphosphate (48). More recently, a computational study of serum amyloid A fibrils interacting with heparin has provided evidence that extra densities in cryo-EM structures represent bound heparin/HS (49). Tau amyloid fibrils have been found to colocalize with different cofactors. In AD, tau fibrils were observed to colocalize with HS/HSPGs (17, 18, 51), RNA (52), and a number of lipids (53). Cryo-EM maps for tau PHF and SF fibrils found in AD have been resolved with multiple regions of additional densities surrounding the fibril cores (Fig. 2).

**Figure 2:**
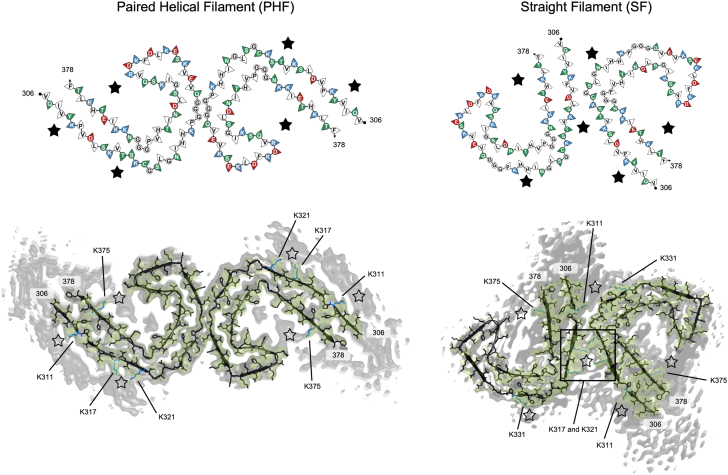
**Cross-sections of tau PHF and SF cryo-EM *ex vivo* structures.** Residues 306 to 378 form the fibril core, and the remaining residues are intrinsically disordered. Residues are colored based on their properties: *red* (acidic), *blue* (basic), *green* (polar), and *white* (nonpolar). *Stars* indicate the location of candidate heparin-binding sites for both polymorphs based on the locations of unidentified extra densities in cryo-EM maps of *ex vivo* tau PHF (PDB code: 5O3L) and SF (PDB code: 5O3T) fibrils from Fitzpatrick *et al.*, 2017 (40). The cryo-EM sharpened density of the fibril structure is represented in *green* with a contour level of 4.5*σ*, and unsharpened densities are represented in *gray* with a contour level of 4*σ*. Figures were generated using FibMap (49) and PyMOL (55). PDB, Protein Data Bank; PHF, paired helical filament; SF, straight filament.

Sampling the interaction of heparin with all possible binding sites on the tau fibril using detailed all-atom molecular dynamics (MD) simulation involving explicit solvent is currently impractical. This is due to the large volume of the overall system, on the order of half a million atoms for a minimal fibril model, and the relatively slow time scale for diffusing motion of the heparin ligand. An alternative computational approach is offered by Brownian dynamics (BD). Two key approximations are made. First, a simplified model of the solvent is employed in which the ion concentration and dielectric properties of water are used in continuum-based models for computation of the electrostatic forces between the macromolecules. This dramatically reduces the number of atoms in the model. Second, the motion of atoms is assumed to be overdamped and diffusive, represented as a random walk with no linear or angular velocities (54). In BD, atomic positions are updated with significantly larger time steps, up to 1000 times larger than those used in MD simulations, in which fast inertial motions are effectively ignored. As a result, for systems in which a significant effort must be made to simulate relatively few replicas using MD, the BD method can be used to generate thousands of trajectories.

In this work, we employ a multiscale simulation methodology that combines the strengths of the BD and MD simulation approaches (49, 54). As it has been well established that the interactions between heparin and tau are primarily electrostatic, understanding the diffusive dynamics for a large system using BD is an appropriate method for this system. However, to properly refine the resulting putative bound state complexes, we used detailed all-atom MD simulations to refine the structures and provide a detailed atomistic description of the bound states. We used 10 different poses of heparin and 20 tau fibril conformations for a total of 200 representations of the system. For each representation, 1000 BD trajectories were generated. From the BD simulations, 10 putative bound state structures were identified for each candidate binding site and refined using all-atom MD simulations. Our multiscale approach allows us to exhaustively explore and identify putative binding sites using BD simulations and refine those binding poses using MD simulations.

## Results

### Electrostatic steering directs heparin binding to K375–R349 in tau PHF

**Figure 3:**
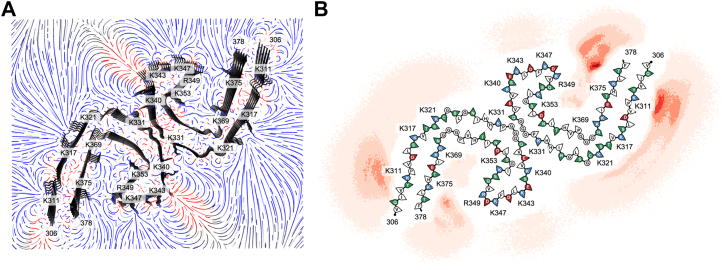
**Sampling of heparin binding modes to tau PHF.***A,* electrostatic field of tau PHF (*top–down view*) computed by APBS to guide BD simulations. *Blue* indicates positive charge, *red* indicates negative charge, and *gray* indicates neutral. *B,* density of the final positions of the heparin sulfur atoms from BD trajectories. APBS, Adaptive Poisson–Boltzmann Solver; BD, Brownian dynamics; PHF, paired helical filament.

All possible binding sites were defined as basic residues exposed to solvent that correspond to positive regions of the electric field of the fibril (Fig. 3*A*). The results of the BD simulations for heparin bound to the tau PHF core are shown as a density map (Fig. 3*B*) and the probabilities calculated in Table 1. Those sites with a low number of binding events (≤2%) and that proximate to a negatively charged solvent-exposed residue (K331 across E342 in PHF) or immediately next to a negatively charged residue (K340 near E338, K343 next to E342 and near D345, K347 next to D348 and near D345, and R349 next to D348 in both tau polymorphs) were not further considered for MD simulations as it would be unlikely for heparin to bind to these sites because of the negatively charged electrostatic interference that would occur when heparin approaches these sites. Therefore, our all-atom MD simulations used binding conformations of the fibril–heparin complexes for sites K311, K317, K321, and K375. These sites that correspond to excess densities in the cryo-EM map were validated by false discovery rate analysis (56) (Fig. S1). The densities near these residues were visible at a high threshold, indicating that the probability of these densities being noise was insignificant.

**Table 1:** Probability of heparin binding to tau PHF

Residue	Heparin bound (no. of trajectories)	Heparin bound (%)
K311∗	48,463	24.2 ± 0.1
K317∗	10,443	5.22 ± 0.05
K321∗	14,146	7.07 ± 0.06
K331	3757	1.88 ± 0.03
K340	628	0.31 ± 0.01
K343	3409	1.71 ± 0.03
K347	1158	0.58 ± 0.02
R349	1219	0.61 ± 0.02
K353	1723	0.86 ± 0.02
K369	3452	1.73 ± 0.03
K375∗	52,415	26.2 ± 0.1

Number of trajectories and percentages for heparin bound to each solvent-exposed positively charged binding site from BD simulations. Residues denoted by an asterisk were selected for further refinement of binding modes through MD simulations. Errors were computed using a binomial proportion confidence interval where the observed binomial proportion is the fraction of heparin bound.

The tau fibril core contains many regions of positive charge, interspersed by a few negatively charged residues, which define the electric field of the fibril that serves to electrostatically steer heparin away from acidic residues and toward ladders of basic residues. Due to the large positively charged electric field, heparin can misalign from the principal axis of the fibril at the target site and end up perpendicular or completely dissociate to a neighboring binding site. This suggests a heparin–fibril interaction driven largely by electrostatic steering.

**Figure 4:**
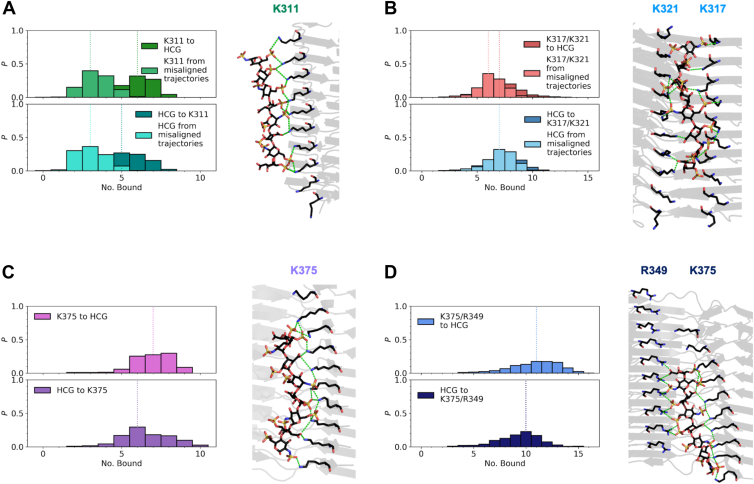
**Refinement of heparin binding modes to tau PHF core through MD simulations.** Probability of a contact between a unique NZ atom of a lysine or NH1/NH2 atom of an arginine of (*A*) K311, (*B*) K317–K321, (*C*) K375, and (*D*) K375–R349 to a HCG (*top*) and probability of a contact between a unique HCG atom to the respective basic residues *(bottom*). Lighter distributions correspond to misaligned heparin trajectories. *Dotted lines* represent the median number bound. A representative image for each bound pose, which represents the total number of possible contacts between HCG and the NZ atom of a lysine or NH1/NH2 atom of an arginine, is shown. HCG, heparin charged group; MD, molecular dynamics; PHF, paired helical filament.

Of the 10 trajectories with heparin initially bound to K311, only seven trajectories end with heparin still bound to K311 for the PHF polymorph. In the remaining trajectories, heparin ends perpendicular to the principal axis of the fibril. These misaligned trajectories resulted in a median of three K311 residues bound to a heparin charged group (HCG) throughout the trajectories, and a median of three HCGs bound to a K311 residue (Fig. 4*A*). For the trajectories where heparin remains bound to K311, a median of six K311 residues is bound to an HCG, and a median of five HCGs is bound to a K311 residue.

K317 and K321 will be considered as one binding site because of the proximity of these residues and how the side chains are angled toward each other, suggesting that both sites simultaneously play a role in stabilizing heparin. Of the 20 trajectories with heparin initially bound to K317–K321 in the PHF, 15 trajectories end with heparin bound to K317–K321, and the remaining five trajectories end with heparin misaligned from the principal axis of the fibril. For the PHF, where heparin remains bound to K317–K321, a median of seven K317–K321 residues is bound to HCG (Fig. 4*B*). For the trajectories where heparin is misaligned, a median of six K317–K321 residues is bound to HCG. A median of seven HCGs bound to K317–K321 is observed for heparin-bound and misaligned trajectories.

From the trajectories with heparin initially bound to K375, heparin remained bound for all 10 trajectories. Approximately 88% of the conformational ensemble involves an HCG in contact with both K375 and R349, indicating that R349 participates in secondary interactions with heparin. Considering only those frames with heparin in contact with K375, a median of seven K375 residues is bound to HCG and a median of six HCGs is bound to a K375 residue (Fig. 4*C*). Frames that include HCG in contact with K375 and R349 have a median of 11 K375–R349 residues bound to HCG and a median of 10 HCGs bound to K375–R349 (Fig. 4*D*).

Considering the largest number of heparin contacts possible with a binding site, approximate binding energies for tau fibril–heparin complexes are ordered from weakest to strongest as K311 < K317–K321 < K375 < K375–R349 (Fig. S4*A*). The probability of heparin bound to K375 from BD simulations (26.2 ± 0.1%) and the relatively strong binding energy indicate that K375 could potentially be a primary binding site for heparin. K311 also has a high probability that heparin is bound (24.2 ± 0.1%). However, K311 has the lowest binding energy of all four sites, suggesting that K311 is a secondary binding site for heparin. There is a lower probability for heparin to bind to K317 (5.22 ± 0.05%) and K321 (7.07 ± 0.06%) compared with K311 and K375, yet heparin interactions with both K317–K321 have a stronger binding energy than K311.

### Electrostatic steering directs heparin binding to K375–R349 in tau SF

**Figure 5:**
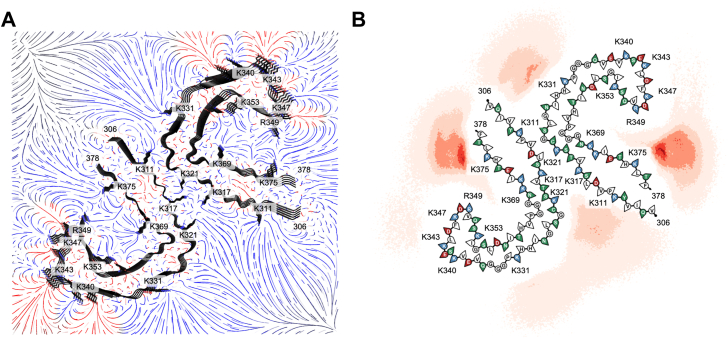
**Sampling of heparin binding modes to tau SF.***A,* electrostatic field of the tau SF (*top–down view*) computed by APBS to guide BD simulations. *Blue* indicates positive charge, *red* indicates negative charge, and *gray* indicates neutral. *B,* density of the final positions of the heparin sulfur atoms from BD trajectories. APBS, Adaptive Poisson–Boltzmann Solver; BD, Brownian dynamics; SF, straight filament.

**Figure 6:**
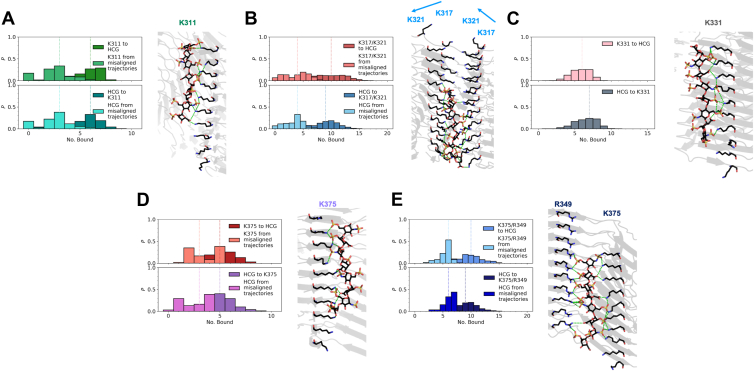
**Refinement of heparin binding modes to the tau SF core through MD simulations.** Probability of a contact between a unique NZ atom of a lysine or NH1/NH2 atom of an arginine of (*A*) K311, (*B*) K317–K321, (*C*) K331, (*D*) K375, and (*E*) K375–R349 to a HCG (*top*) and probability of a contact between a unique HCG atom to the respective basic residues (*bottom*). The *blue arrows* in (*B*) represent the directionality of the residue arrangement in the protofilaments. Lighter distributions correspond to misaligned heparin trajectories. *Dotted lines* represent the median number bound. A representative image for each bound pose, which represents the total number of possible contacts between HCG and the NZ atom of a lysine or NH1/NH2 atom of an arginine, is shown. HCG, heparin charged group; MD, molecular dynamics; SF, straight filament.

Results from BD simulations of heparin binding to the tau SF fibril core are shown in Figure 5*B*, along with the binding site probabilities (Table 2). Binding conformations of the fibril–heparin complexes for sites K311, K317, K321, K331, and K375 were used in all-atom MD simulations for the tau SF fibril core. False discovery rate analysis of the densities near these residues at a high threshold shows that these densities are a true signal rather than noise (Fig. S2). Heparin remained bound to K311 in 4 of 10 trajectories, with heparin initially bound to K311. Heparin moved to bind at K317–K321 in one trajectory. The remaining five trajectories resulted in heparin not aligned with the principal axis of the fibril and ended up in contact with K317–K321. There is a median of three K311 residues bound to HCG and a median of three HCGs bound to K311 for the trajectories in which heparin is misaligned (Fig. 6*A*). For the trajectories where heparin remains bound to K311, there is a median of six K311 residues bound to HCG and a median of six HCGs bound to a K311 residue. Due to the arrangement of protofilaments in the SF polymorph, the intensity of the electric field from the positively charged region of K317–K321 is greater than the electric field intensity observed in the PHF polymorph. This causes our heparin model to more likely misalign from the principal axis of the fibril of the SF polymorph because of this greater contribution of positive charge to the K317–K321 region.

**Table 2:** Probability of heparin binding to tau SF

Residue	Heparin bound (no. of trajectories)	Heparin bound (%)
K311∗	21,006	10.50 ± 0.07
K317∗	20,711	10.36 ± 0.07
K321∗	13,329	6.66 ± 0.06
K331∗	15,939	7.97 ± 0.06
K340	116	0.058 ± 0.005
K343	1310	0.66 ± 0.02
K347	1313	0.66 ± 0.02
R349	111	0.056 ± 0.005
K353	128	0.064 ± 0.006
K369	4071	2.04 ± 0.03
K375∗	50,771	25.4 ± 0.1

Number of trajectories and percentages for heparin bound to each solvent-exposed positively charged binding site from BD simulations. Residues denoted by an asterisk were selected for further refinement of binding modes through MD simulations. Errors were computed using a binomial proportion confidence interval where the observed binomial proportion is the fraction of heparin bound.

For the trajectories with heparin initially bound to K317–K321, 13 of 20 trajectories end with heparin bound to K317–K321, six trajectories end with heparin misaligned from the principal axis of the fibril, and one trajectory completely dissociates from K317–K321 to K311. When heparin remains bound to K317–K321, a median of 10 K317–K321 residues is bound to HCG and a median of 9 HCGs are bound to K317–K321 (Fig. 6*B*). From misaligned heparin trajectories, a median of four was obtained for both cases of K317–K321 residues bound to HCG and HCGs bound to K317–K321. The 10 trajectories in which heparin was originally bound to K331 remained bound. A median of six K331 residues is bound to HCG, and a median of seven HCGs is bound to K331 (Fig. 6*C*).

As observed for the tau PHF core, for the trajectories initially bound to K375, approximately 88% of the conformational ensemble involves heparin in contact with both K375 and R349. Of the 10 trajectories with heparin originally bound to K375, only one trajectory ended with heparin lateral to the principal axis of the fibril. Considering the frames with heparin in proximity to K375 only, the median number of K375 residues bound to HCG and HCGs bound to K375 is five (Fig. 6*D*). There is a median of three K375 residues bound to HCG and for HCGs bound to K375 observed in the frames of the trajectory where heparin is misaligned from the K375 residue array. Frames with heparin bridging K375 and R349 have a median of 10 K375–R349 residues bound to HCG and a median of nine HCGs bound to K375–R349 (Fig. 6*E*). The frames of the misaligned heparin trajectory that involved contacts with K375 and R349 have a median of six K375–R349 residues bound to HCG and a median of six HCGs bound to K375–R349.

Taking into account the largest number of heparin contacts possible with a binding site, approximate binding energies for tau fibril–heparin complexes are ordered from weakest to strongest as K311 < K375 < K331 < K375–R349 < K317–K321 (Fig. S4*B*). The SF protofilament arrangement contains four basic residue arrays of K317 and K321, which may be why heparin has a more energetically favorable preference for this site compared with the PHF polymorph. However, K317–K321 may not be a primary binding site for heparin because of the lower probability of heparin being bound to K317 (10.36 ± 0.07%) and K321 (6.66 ± 0.06%). K375 is likely a primary binding site for heparin with secondary interactions from R349 to further stabilize the fibril–heparin complex, as evidenced by K375 having the highest probability of heparin being bound (25.4 ± 0.1%) and the relatively strong binding energy. K311 and K331 are more likely to be secondary binding sites for heparin because of the lower likelihood of heparin binding to these sites (10.50 ± 0.07% and 7.97 ± 0.06%, respectively) and less favorable binding energies compared with K375–R349.

**Figure 7:**
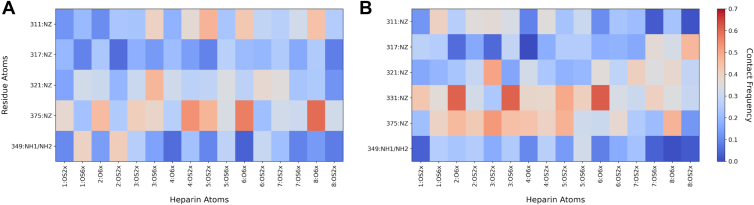
**Heparin atom group contact frequencies at tau fibril binding sites.** Ensemble-averaged contact frequency maps between grouped heparin terminal oxygen atoms and basic side-chain atoms of the PHF (*A*) and SF (*B*) fibril cores. Heparin atoms were grouped by monosaccharide units and functional group (sulfate OS2X/OS6X or carboxylate O6X): odd numbers correspond to glucosamine and even numbers to iduronic acid. Protein contacts were calculated by summing heparin contact frequencies with NZ (lysine) and NH1/NH2 (arginine) across all segments of the fibril within each unit of heparin using a 4.5 Å interaction distance cutoff. PHF, paired helical filament; SF, straight filament.

Some lysines consistently show higher contact scores with certain heparin sulfates. Other residues have weaker or more scattered contacts, indicating more dynamic or transient interactions with heparin. In Figure 7*A*, K375 is the site most involved in the binding of heparin to PHF fibrils, specifically with the iduronic acid groups in residues 4, 6, and 8. The higher contact frequency within K349, particularly in the sulfate group OS6X of the glucosamine within the first unit of heparin and the sulfate group OS2X of the iduronic acid within the second unit of heparin, shows that R349 stabilizes interactions with heparin when bound to K375. However, these interactions are less prominent within the SF fibril core, suggesting that R349 is spatially less accessible. Prominent contacts are observed within the range of the first five units of K375 (Fig. 7*B*). The consistent span of contacts from K375 across these units shows that this residue engages in stabilizing a stretch of adjacent sugars, suggesting that this particular site serves as a principal anchoring site in the ensemble. K331 is observed to have the highest contact frequencies with certain heparin sulfates (O6X in unit 2, OS6X in unit 3, and O6X in unit 6), showing strong but localized contacts compared with K375. K375 likely forms multiple simultaneous salt bridges with neighboring sugars and adopts conformations that allow heparin to flexibly interact along a stretch of heparin, further supporting that K375 is a primary binding site in both PHF and SF fibril polymorphs.

## Discussion

Our results from BD and MD simulations show that heparin binding is defined by electrostatic interactions (Figs. 3 and 5). The negatively charged residues immediately surrounding positively charged residues (K331 in PHFs, K340, K343, K347, and R349) electrostatically steer polyanionic heparin to be bound to positively charged residues that are not impeded by the electrostatic field of a negatively charged residue. When heparin is bound to a basic residue array, salt bridges formed between the HCGs and side chains of lysines and arginines stabilize the heparin conformations (Figs. 4 and 6). Both the tau PHF and SF polymorphs have a primary binding site at K375–R349 from our simulations. Although it was unlikely for heparin to be bound to R349 alone from BD simulations (PHF: 0.6% and SF: 0.06% from Tables 1 and 2), MD simulations of the initial binding poses of heparin within the cutoff distance of K375 revealed that R349 secondary interactions are prominent, suggesting that heparin exhibits a more stable conformation from interacting with both these residues (Fig. S4, *A* and *B*). From the molecular mechanics Poisson–Boltzmann surface area calculations, the relationship between the binding energy and the number of contacts is approximately linear; each additional contact with an HCG stabilizes the tau fibril–heparin complex by approximately −1.6 ± 0.1 to −3.2 ± 0.2 kcal/mol. For K375 in PHF fibrils, each additional contact with an HCG contributes −3.2 ± 0.2 kcal/mol, compared with −2.9 ± 0.3 kcal/mol in SFs. This suggests stronger or more favorable contacts between heparin and K375 in PHF fibrils. The heparin contacts with K375–R349 contribute −2.7 ± 0.1 kcal/mol in PHF and −2.2 ± 0.1 kcal/mol in SF polymorphs. The strongest contribution from additional contacts with HCGs is observed in K331 with −3.1 ± 0.1 kcal/mol in SFs. These observations are consistent with estimates of stabilizing energies of −3 to −5 kcal/mol for individual salt bridges (57).

The atomistic detail revealed by this study includes the identification of chief contacts between sulfate groups of heparin and basic residues of the tau fibrils, parallel or perpendicular heparin binding poses with respect to the fibril axis, and specific binding sites that are predominant from our calculations. The refined binding poses from MD simulations give detailed descriptions of the favorable contacts involved in the fibril–heparin complex.

### Role of salt in tau fibril assembly and stabilization

The formation of tau fibrils *in vitro* depends on the identity of the salt in phosphate buffer that defines the final structure of the fibril. AD PHF fibrils were formed in the presence of magnesium chloride (58), and the chronic traumatic encephalopathy protofilament fold was formed with sodium chloride (38). We did not consider the identity of the salt in our simulations. To investigate the role of salts in our all-atom simulations, we computed mass–density volumetric maps (1.0 Å resolution) averaged over all frames within a trajectory replicate for a specific binding site on both protofilaments (Figs. S5–S8). These volumetric maps are visualized as isosurfaces. As expected, chlorine ions accumulate near surrounding positively charged residues within the tau fibril core when heparin is bound to a specific site, consistent with the strong densities observed in the maps. Sodium ion densities also follow expected trends, localizing near negatively charged residues and near HCGs that are not directly coordinated to residues. Notably, the most pronounced sodium densities are found buried within the fibril core near the terminal ends, where residues D314 and D372 are not solvent exposed; these densities also appear in proximity to bound heparin. A particularly interesting observation in the SF protofilament is that when heparin binds at K317–K321, a strong chloride density remains at this site—even though the HCGs are not fully interacting with all the residues along the K317–K321 site—indicating continued chloride interactions with these lysines (side view) (Fig. S7). In addition, sodium densities in the SF appear only near negatively charged residues in the fibril core, and no sodium density is observed near heparin at the K317–K321 site (Fig. S8).

These results suggest that the electrostatic attraction between chloride and HCGs with K317–K321 in the SF protofilament is stronger than that of other basic residues in the fibril core, likely because of the specific arrangement of the protofilaments. None of the other heparin-binding sites shows a similarly prominent chloride density at a site where heparin is not fully engaged, as we would expect electrostatic repulsion between chloride and HCGs. The presence of both chloride and HCGs at the same site indicates competitive binding with both species, as they both can neutralize the same local positive charge at K317–K321 and heparin binding at this site does not completely displace competing anions. Heparin does not fully saturate the positive charge at K317–K321, so chloride fills in the remaining electrostatic vacancy. This suggests dynamic or partial competition for electrostatic interactions at this site. We do not analyze water contributions to the electrostatics because the electrostatic interactions among HCGs and ions between the basic residues of the fibril core are expected to dominate the local environment.

It remains unclear whether magnesium chloride plays a role in stabilizing tau fibrils, as it has only been reported to be important at the monomer level in forming an AD-specific fold (38, 58). For amyloid-β, the presence of negatively charged GAGs decreases the local salt concentration near the fibril surface (59). Intracellularly, phosphates and magnesium could play a role in stabilizing tau fibrils before they are released into the extracellular space. Once tau aggregates are in the extracellular space, heparin/HSPG interactions stabilize tau fibrils.

### Role of heparin sulfation in tau–heparin binding

Previous experimental work has shown that the degree of sulfation (27) and sulfation pattern (12, 60, 61) of heparin/HSPGs are critical determinants of the strength of the interaction between heparin and tau (62). These factors observed in our BD simulations with varying chain lengths of heparin (tetrasaccharide and decasaccharide) and sulfation patterns (2-O-desulfated and 6-O-desulfated) (Figs. S9 and S10) with tau fibrils were consistent with experimental results. Increasing the chain length, or increasing the degree of sulfation, led to an increased probability for heparin to bind to a site (Table S1). This is observed when sampling heparin binding modes with a chain length of 4 (80,314 unreactive trajectories) compared with a chain length of 8 (59,187 unreactive trajectories). The difference in the number of unreactive trajectories between the 8-mer and 10-mer is negligible because of the length of the fibril extended to 10 layers. For the 10-mer, an extended fibril longer than 10 layers is necessary to provide an ample binding surface for the 10-mer. The short lengths of 4- and 8-mer heparin chains have not shown inhibitory activity for tau; however, longer fragments of 12- and 16-mer had low observed inhibitory activity (62). For the sulfation pattern, 6-O-desulfated heparin was expected to have the most impact on binding, which can be seen with the number of unreactive trajectories with 6-O-desulfated heparin compared with 2-O-desulfated (Table S1).

### Alternative interpretations of extra densities in cryo-EM maps

Tau is known to be hyperphosphorylated in AD (63). Phosphorylation sites have been identified in PHF tau fibrils from AD brain (64, 65). Most phosphorylation sites are located within the unstructured domain of tau. S305 has been identified to be phosphorylated in AD (44), which is more likely to correspond to the extra density in the N-terminal region of the fibril core cryo-EM map than to a bound ligand. More recently, 12 phosphomimetic mutations at specific sites of serine and threonine within the “fuzzy coat” induce the *in vitro* assembly of full-length tau into PHF fibrils that resemble the same structure as the *ex vivo* PHF fibrils (66). These findings indicate the role of site-specific phosphorylation in the “fuzzy coat” of monomeric tau already disassembled from microtubules, with the microtubule-binding regions of tau remaining unmodified, in facilitating spontaneous assembly of PHF fibrils (66). As such, the additional densities surrounding certain residues in the tau fibril core may result from post-translational modifications rather than heparin binding.

Cofactors can be incorporated in tau fibrils through binding to the fibril core. This can act to stabilize the positively charged residue arrays at the protofilament interface and assist in the prion-like mechanism of spreading and intracellular seeding of tau aggregates in neighboring cells. An important experimental finding supporting this conjecture is that extracellular tau fibrils, but not monomers, were found to be internalized in cells (23, 67) by binding to HSPGs and tau fibril uptake was blocked by binding to heparin (22). Due to the different outcomes in the propagation of tau fibrils upon binding to heparin and HSPGs, HSPGs may exhibit different binding behavior to tau fibrils compared with what was observed for heparin in this study.

## Conclusion

In this work, we employed a multiscale simulation methodology to provide the first *de novo* computational predictions of sites for heparin binding to tau fibrils. We demonstrate that heparin interactions with tau are dictated by long-range electrostatic interactions between unobstructed basic residues in the tau fibril core and negatively charged groups within the heparin polyanion. Our results indicate that K375 is the primary site for heparin binding to tau fibrils. In addition, we find that binding to K375 is further stabilized through secondary interactions with R349. The predictions of our simulations were critically compared with cryo-EM density maps. The proposed heparin-binding sites resulting from our simulation study are consistent with the observed excess densities surrounding K375 in the cryo-EM map and the fact that HS codeposits with tau fibrils in AD. Taken together, our results support the identity of the unknown density near K375 as a bound and negatively charged GAG.

In addition to the identification of heparin-binding sites, our multiscale simulations provide insight into the mechanism of heparin binding to tau fibrils. Electrostatic funnels formed by arrays of lysine and arginine residues adjacent to negatively charged residues act to steer highly anionic heparin toward positively charged residue arrays on the fibril surface. Subsequent binding is reinforced by salt bridges that involve the charged groups on heparin and side chains of the Lys and Arg residues. The mechanism is consistent with conformational selection by the heparin substrate of potential binding sites, followed by refinement of fibril side chains to refine the bound state. This mechanism shares features of the dock-and-lock mechanism observed in amyloid fibril elongation (68, 69, 70, 71).

Taken together, our findings provide a structural framework for understanding the location and mechanism of the binding of heparin to tau fibrils, which is known to inhibit the uptake of tau fibrils by neighboring cells. Building on these findings, future computational work can explore the nature of HS interactions with tau fibrils, in comparison to heparin, to better understand the distinct roles these glycans play in the stabilization and propagation of tau aggregates.

## Experimental procedures

Trajectories were analyzed using MDAnalysis (72, 73), a Python library fiscally sponsored by NumFOCUS. Fibril images were rendered using PyMOL from Schrödinger, Inc. (55). The methods were adapted from previous work on the binding of heparin to SAA fibrils (49) and applied here to tau fibrils.

### Fibril models for BD

The CHARMM-GUI Protein Data Bank [PDB] reader and manipulator was used to add hydrogens and neutralize both N- and C-terminal ends in each peptide in the five-layer fibril core (residues 306–378) of the PHF (PDB code: 5O3L) and SF (PDB code: 5O3T) tau polymorphs (40, 74). Fibrils were extended to 10 layers to provide a fibril surface area sufficient for heparin octasaccharide binding (described in supporting information). The PHF nucleus has previously been stated to consist of 8 to 14 tau monomers (6).

Each fibril model was solvated using the TIP3P water model and 150 mmol NaCl and simulated in GROMACS 2021.5 using the CHARMM36m force field at 310.15 K (75, 76). A rhombic dodecahedron box was defined with a minimum of 2 nm from the box edge to prevent interperiodic self-contact of the fibril. Energy minimization was performed with the steepest descent algorithm. Three 5 ns equilibrations of the fibril structures were performed with a 1 fs timestep: (1) An NVT ensemble was sampled with position-restraining forces applied to the backbone (400 kJ mol^−1^ nm^−2^) and side chains (40 kJ mol^−1^ nm^−2^) using the velocity-rescaling thermostat (77). (2) An NPT ensemble was sampled with the same restraints using the velocity-rescaling thermostat and Berendsen barostat (77, 78). (3) An NPT ensemble was sampled without position restraints using the Nose–Hoover thermostat and Parrinello–Rahman barostat (79, 80, 81). Simulations for each fibril model were performed for 500 ns with a 2 fs timestep using the same thermostat and barostat in the third equilibration.

### Heparin model for BD

The heparin octasaccharide molecule, 2-O-sulfo-*α*-l-iduronic acid-[1 → 4]-*α*-*N*-sulfo-d-glucosamine-6-O-sulfate (*α*-L-IdoA(2S)-[1 → 4]-*α*-D-GlcNS(6S)) was constructed using the CHARMM-GUI glycan reader and modeler (74, 82). The heparin molecule was solvated using the TIP3P water model and 150 mmol NaCl and simulated in GROMACS 2021.5 using the CHARMM36m force field (75, 76). A cubic box was defined with a minimal distance of 1.5 nm from the edge of the box. Energy minimization was performed with the steepest descent algorithm. The first equilibration in the NVT ensemble was performed for 2 ns with position-restraining forces on the glycan rings (400 kJ mol^−1^ nm^−2^), nonring substituents (40 kJ mol^−1^ nm^−2^), and glycan ring dihedrals (4 kJ mol^−1^ nm^−2^). The second equilibration was performed for 2 ns without position restraints using the velocity-rescaling thermostat with a timescale of 0.2 ps and Berendsen barostat with a timescale of 2 ps (77, 78). Both equilibrations used a 1 fs timestep. A production run was conducted for 1 μs with a 2 fs timestep using the same thermostat and barostat in the second equilibration.

### BD of heparin–fibril binding

Frames from the MD trajectory of the fibrils and heparin were organized using agglomerative clustering with Ward’s minimum variance metric (83). Clustering was performed in Julia using the Clustering.jl package (84). For the tau PHF and SF, clustering was based on the positions of the NZ atom of K317 and K321. To maintain computational efficiency, clustering of the fibril structures was limited to two possible heparin-binding sites. Further subdivision of all potential binding sites was neither expected to significantly affect the characterization of heparin-binding behavior nor provide additional statistically distinct fibril states, as the experimentally derived tau fibril structures are thermodynamically and kinetically stable with relatively small structural fluctuations. For heparin, clustering was done on the basis of the positions of the sulfur atoms. We selected 10 clusters for each lysine in each fibril polymorph for a total of 20 clusters for each fibril polymorph and 10 clusters for heparin to obtain a broad selection of conformations from the conformational ensembles as starting structures for BD simulations. The medoid frame in each cluster was used as an input structure for BD simulations, and electrostatics were calculated with the Adaptive Poisson–Boltzmann Solver at 310.15 K (85).

BD simulations were performed using the BrownDye2 simulation program, employing the Northrup–Allison–McCammon algorithm at 310.15 K (54). The parameters for the solvent include the dielectric constant of 78 and the relative viscosity of 1. The smallest possible time step size for a system with only rigid cores and frozen chains is 1.0 ps, and the minimum step size used in situations when the heparin ligand approaches a potential fibril binding site is 0.01 ps. The reaction criteria were defined as follows: a minimum of three residue-heparin contacts must be formed with a distance threshold of 7.5 Å between the reference atoms. For heparin, the reference atoms were S2 and S6 of each alphaD-GlcNS(6S) and S2 of each alpha-l-IdoA(2S). For both tau fibril polymorphs, reference atoms for arginine and lysine were defined to be the CZ atom or NZ atom, respectively, in all solvent-exposed basic residues within the fibril core: K311, K317, K321, K331, K340, K343, K347, R349, K353, K369, and K375. For each fibril–heparin pair from the input structures, 1000 trajectories were generated. A total of 200,000 trajectories were generated for each fibril polymorph. All other defined parameters not stated here were set to the default values.

### MD of heparin–fibril complexes

Final frames from BD simulations ending with heparin bound to either K311, K317, K321, or K375 for both tau fibril polymorphs (and K331 for SF) were used as starting configurations for MD simulations if the heparin orientational angle was ≤ 45° relative to the principal axis of the fibril. The fibril axis was defined by the vector from the centroid of the third layer to that of the third-to-last layer and the heparin axis by the vector from the first to the last ring centroid. For each binding site, configurations were grouped using Ward’s minimum variance agglomerative method, with pairwise distance RMSDs calculated from the distances between each heparin sulfur atom and its nearest atom in the binding residue. Based on the results of the BD simulations, only those trajectories successfully bound to K311, K317, K321, and K375 (additionally K331 for SF) were considered for further refinement of the binding modes using MD. Ten clusters for each binding site were obtained, and the medoid configuration for each cluster was selected as the input structure for the MD simulations.

Each starting complex was solvated with the TIP3P water model and 150 mmol NaCl in a dodecahedron box with a minimum distance of 2 nm from the edge of the box. Minimization and equilibration of the complexes were performed using the same parameters as outlined previously in the fibril models section, except that all equilibrations were performed for 1 ns. The production run for each complex was performed for 20 ns with a 2 fs timestep. A fibril side chain or HCG was considered bound if its cationic nitrogen or anionic oxygen was within 4.5 Å of its oppositely charged counterpart. HCGs include oxygens within the sulfate, sulfamido, and carboxylate groups. For our heparin octasaccharide molecule, there was a total of 16 HCGs. We estimated the binding energies of heparin bound to each residue on the fibril for comparison between the residues using the molecular mechanics Poisson–Boltzmann surface area method (detailed in supporting information) (86).

### Data availability

All original data have been presented in the main text or supporting information of this article.

## Supporting information

This article contains supporting information (49, 56, 86, 87, 88).

## Conflict of interest

The authors declare that they have no conflicts of interest with the contents of this article.
