# Supplementary material for: Structural and mechanistic characterization of heparin interactions with tau fibrils
Source: J Biol Chem. 2026 Jan 12;302(3):111153. doi: 10.1016/j.jbc.2026.111153 (PMC12887884; doi:10.1016/j.jbc.2026.111153)
Supplement: Figures S1-S12 and Table S1 [file mmc1.pdf]

**Supporting Information for**

# Structural and mechanistic characterization of heparin interactions with tau fibrils

Fiona Mon and John E. Straub*

Department of Chemistry, Boston University, 590 Commonwealth Ave, Boston, MA, 02215, USA
*Correspondence should be addressed to John E. Straub, straub@bu.edu

**False Discovery Rate (FDR).** False discovery rate (FDR) analysis of cryo-EM maps was performed to determine the significance of the densities by generating confidence maps that can discriminate signal from noise.[56] We used unmasked cryo-EM maps from the deposited two half-maps available in the Electron Microscopy Data Bank (EMD-3741 for PHF and EMD-3743 for SF) to estimate the noise without incorporation of local resolution or atomic model information. Confidence maps are viewed at a threshold of 0.99, which means 99% of the voxels are positive density signals in the map, corresponding to a FDR of 1% (Figure S1 and S2). The probability of these densities being noise is ≤ 1%.

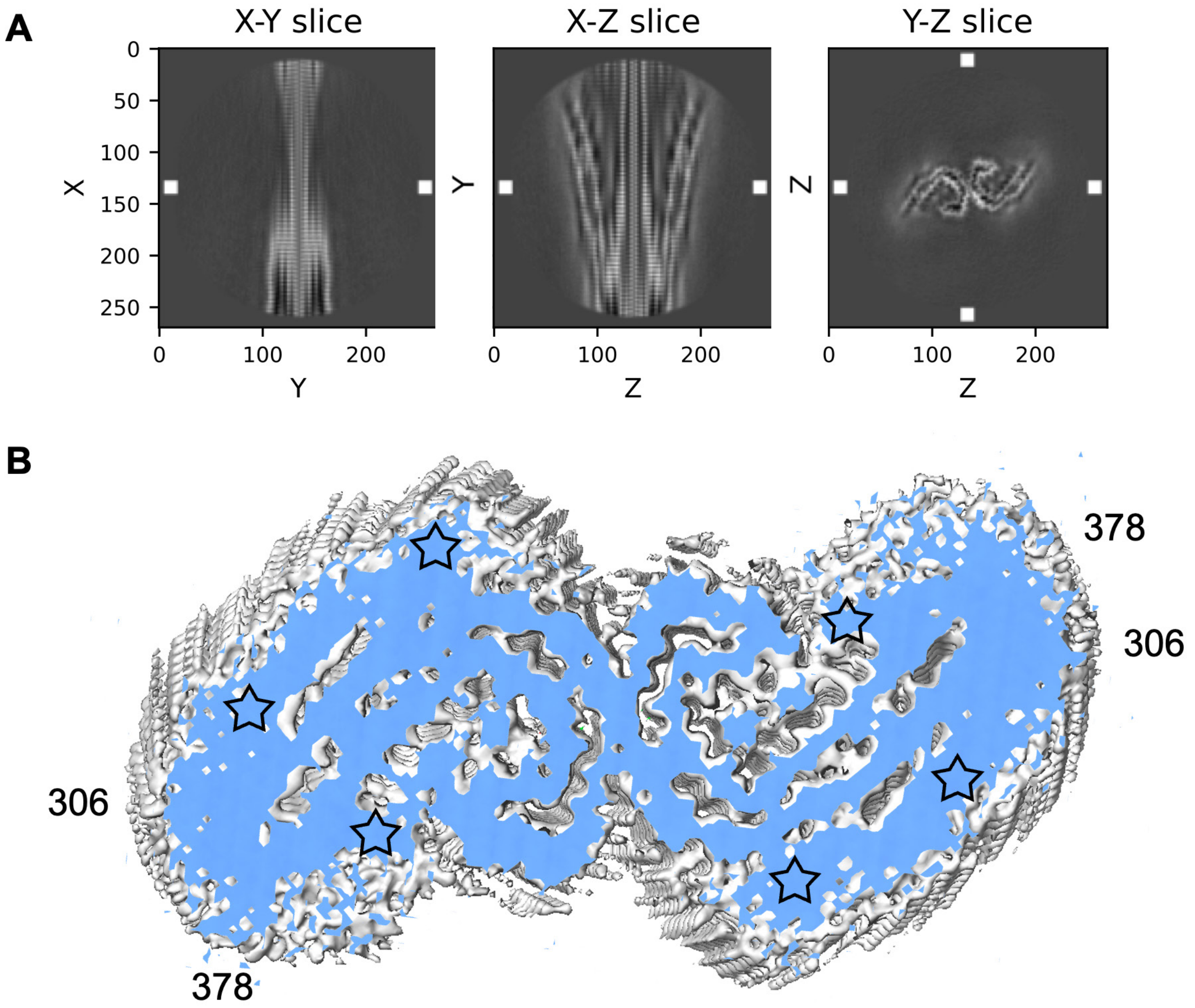


**Figure S1**. False discovery rate (FDR) analysis of PHF cryo-EM map. (A) Slice-views of input PHF unmasked cryo-EM map. White boxes correspond to size of the noise estimation region. (B) PHF confidence map (blue) viewed at a threshold of 0.99 overlayed on the PHF EM map. Stars correspond to our initial predictions of possible heparin binding sites based on the cryo-EM densities.

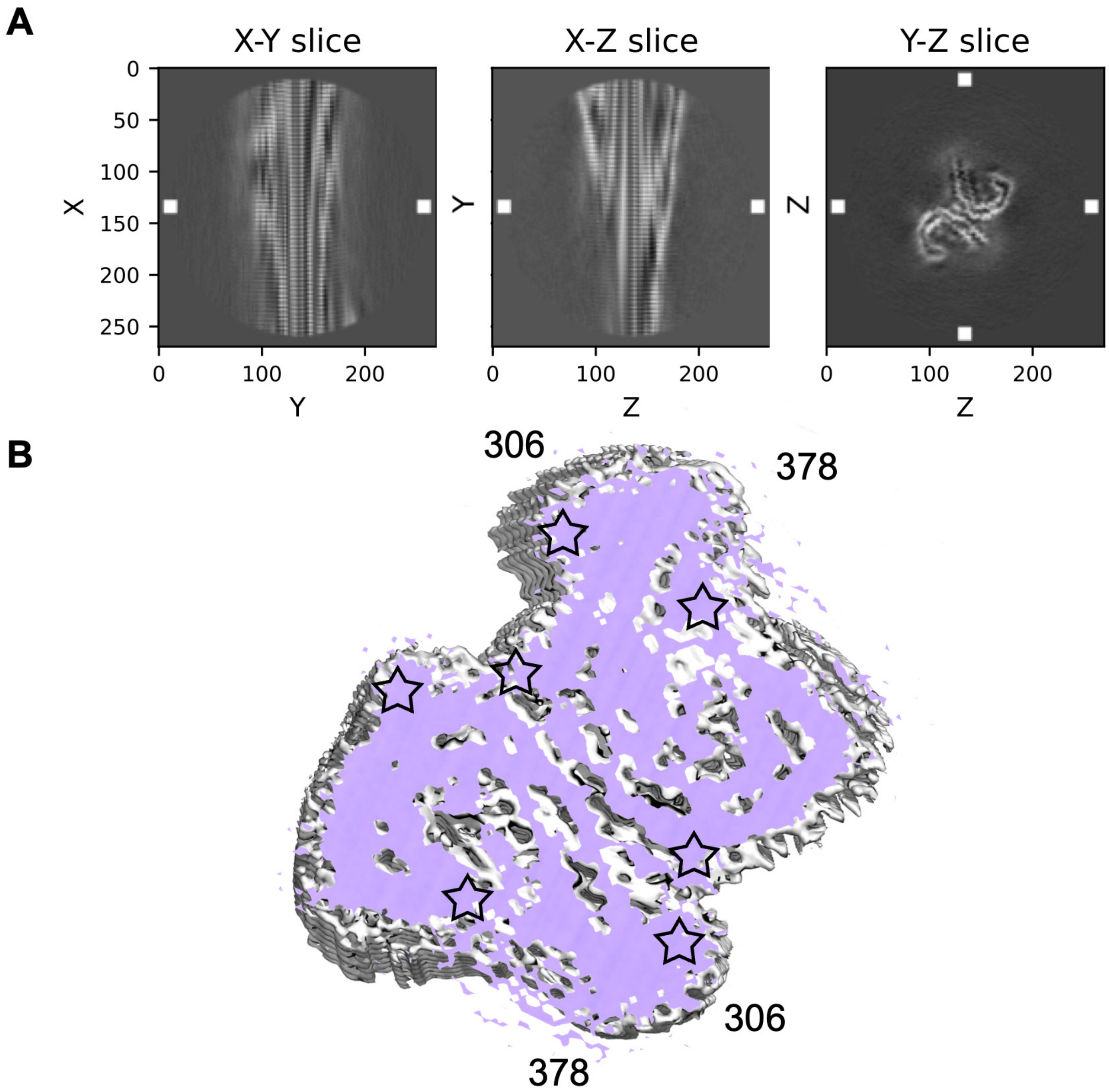


**Figure S2.** False discovery rate (FDR) analysis of SF cryo-EM map. (A) Slice-views of input SF unmasked cryo-EM map. White boxes correspond to size of the noise estimation region. (B) SF confidence map (purple) viewed at a threshold of 0.99 overlayed on the SF EM map. Stars correspond to our initial predictions of possible heparin binding sites based on the cryo-EM densities.

**Extended Fibril Models.** The positions of each atom in the existing layers were used to predict the atomic positions in the next layer according to

$$p_{N_l+1} = \frac{1}{N_l - 2}\left[(2N_l - 3)p_{N_l} - (N_l - 1)p_{N_l-1} + p_1 - p_2\right] \qquad \text{(Eq. S1)}$$

where $p_l$ is the position of an atom in layer $l$ and $N_l$ is the current number of layers in the fibril.[49] This process was repeated to extend each fibril to ten layers. The last layer of the fibril was RMSD-fit using the Kabsch algorithm[87].

**Molecular Mechanics Poisson-Boltzmann Surface Area (MMPBSA).** When calculating the binding free energy from a simulation containing solvated states, large fluctuations from solvent-solvent interactions would comprise the majority of energy contributions. The binding free energy of a protein-ligand complex can be estimated by the Molecular Mechanics Poisson-Boltzmann Surface Area (MMPBSA) method according to the following thermodynamic cycle in Figure S3. The binding free energy can be calculated by:

$$\Delta G_{\text{bind}}^{\text{solv}} = \Delta G_{\text{bind}}^{\text{vac}} + \Delta G_{\text{solv}}^{\text{com}} - \left(\Delta G_{\text{solv}}^{\text{lig}} + \Delta G_{\text{solv}}^{\text{rec}}\right) \qquad \text{(Eq. S2)}$$

where the solvation free energies $\Delta G_{\text{solv}}^{\text{rec}}$, $\Delta G_{\text{solv}}^{\text{lig}}$, and $\Delta G_{\text{solv}}^{\text{com}}$ are calculated by solving the Poisson-Boltzmann equation. $\Delta G_{\text{bind}}^{\text{vac}}$ is given by:

$$\Delta G_{\text{bind}}^{\text{vac}} = \Delta E_{\text{MM}} - T\Delta S \qquad \text{(Eq. S3)}$$

where $\Delta E_{\text{MM}}$ corresponds to the molecular mechanical energy changes. In this study, we neglected the entropic term and computed the effective free energy (referred to as the binding energy in the main text). The MMPBSA calculations were performed with gmx_MMPBSA using the nonlinear Poisson-Boltzmann equation solver.[86] The internal dielectric constant was set to 4.0 and the external dielectric constant was set to 78.0. The total electrostatic energy and forces were computed with the particle-particle particle-mesh procedure with a cutoff distance of 8.0 Å for van der Waals interactions.[88] All other parameters were consistent with system setup in the CHARMM force field or defined by default.

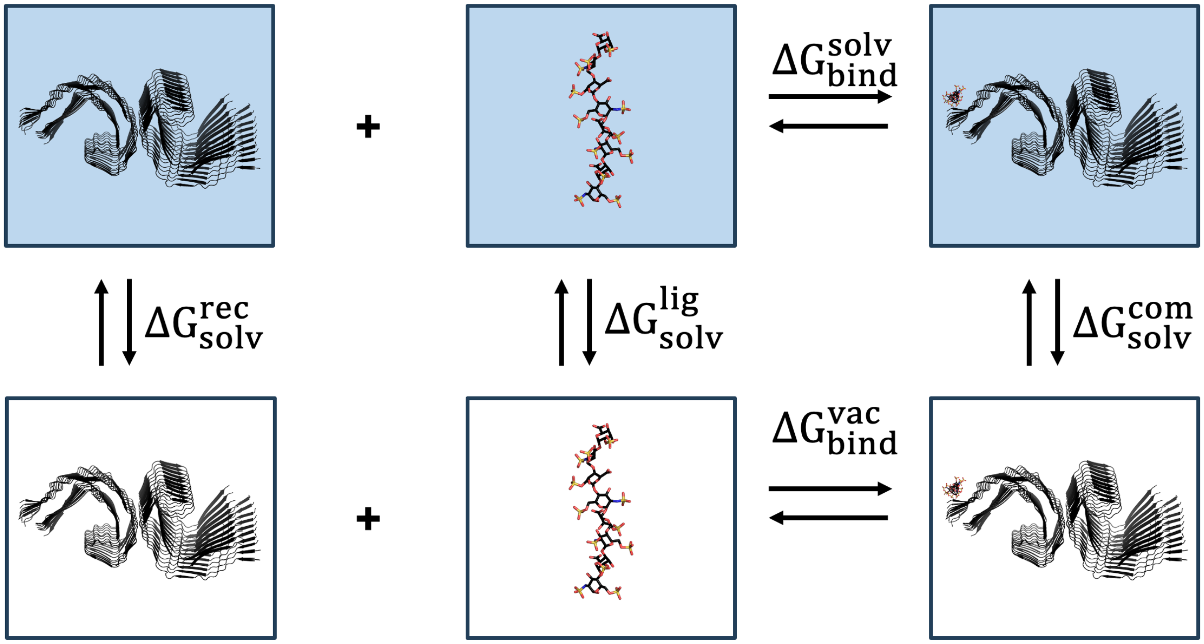


**Figure S3.** Thermodynamic cycle for binding free energy calculations of the fibril-heparin complex (com) where the fibril is the receptor (rec) and heparin is the ligand (lig).

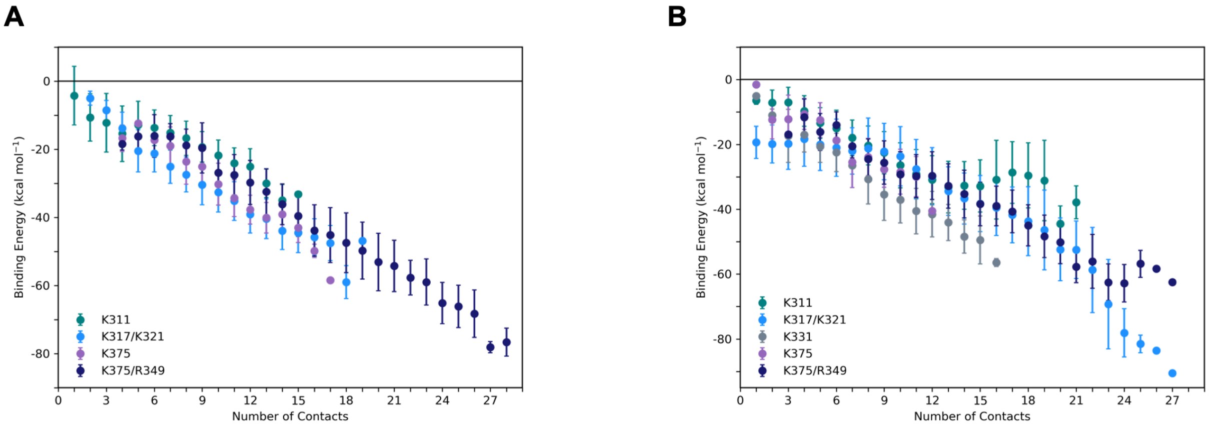


**Figure S4.** Binding energies from MMPBSA of heparin bound to each residue in the (A) PHF and (B) SF.

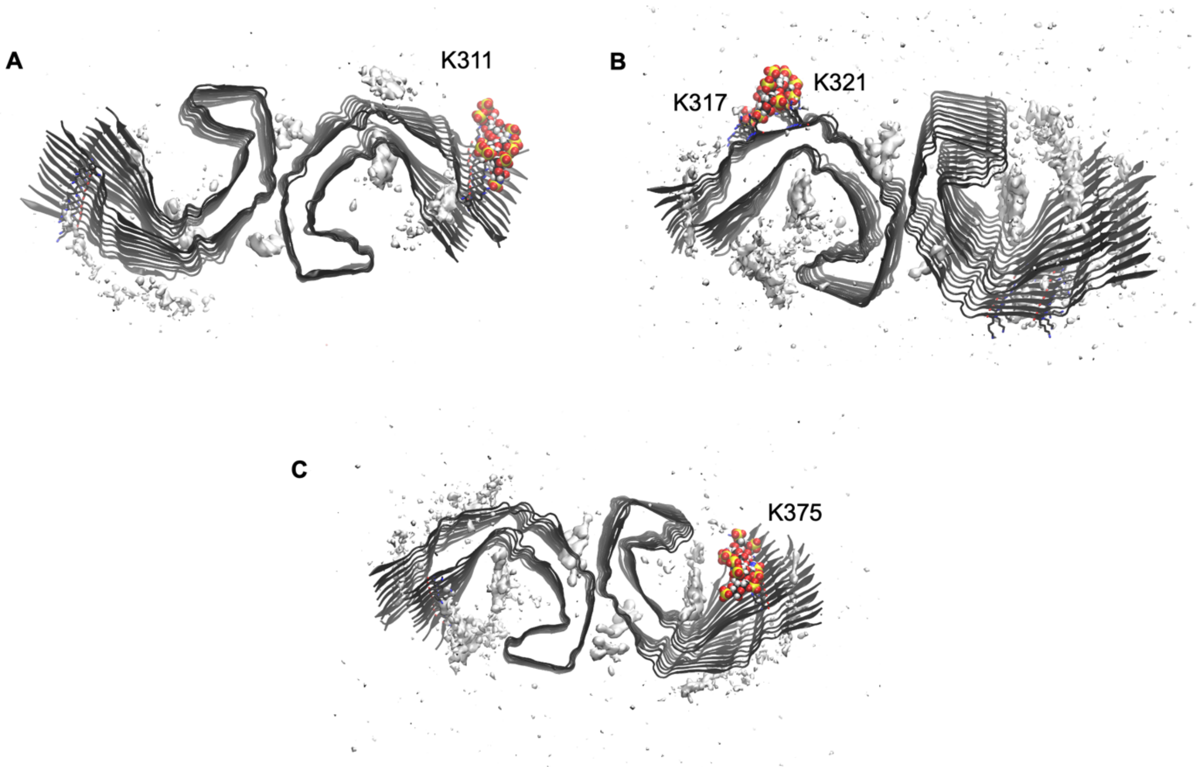


**Figure S5.** Chlorine ion density in tau PHF simulations. Averaged chlorine ion density for one simulation replicate where heparin was bound to (A) K311 (B) K317/K321 and (C) K375. The density representations utilized an isodensity value of 0.047 for K311 and 0.05 for both K317/K321 and K375.

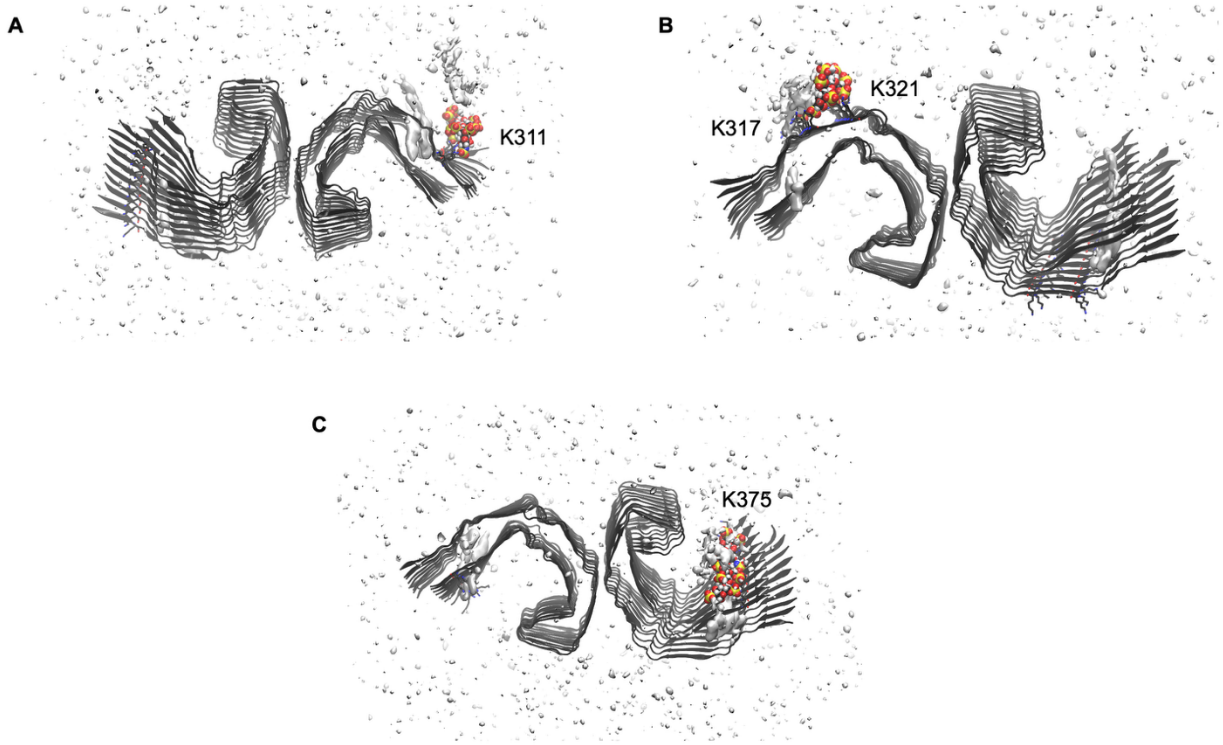


**Figure S6.** Sodium ion density in tau PHF simulations. Averaged sodium ion density for one simulation replicate where heparin was bound to (A) K311 (B) K317/K321 and (C) K375. The isodensity value used in all density maps was 0.023.

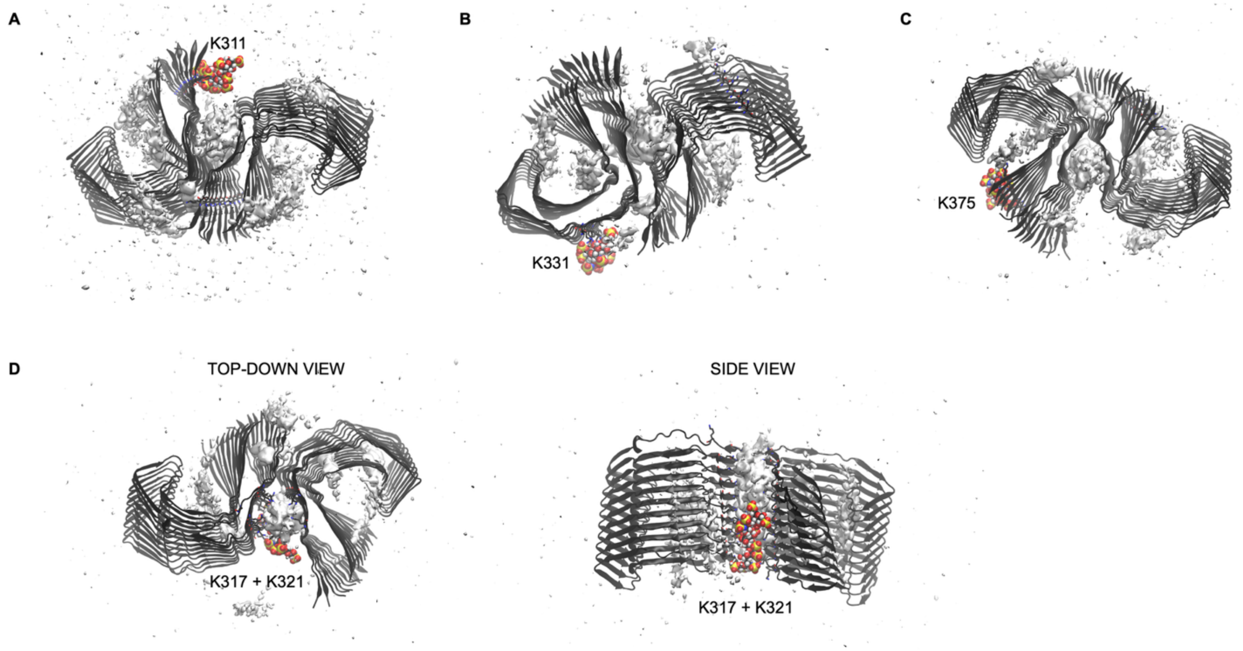


**Figure S7.** Chlorine ion density in tau SF simulations. Averaged chlorine ion density for one simulation replicate where heparin was bound to (A) K311 (B) K331 (C) K375 and (D) K317/K321 with a top-down view (left) and side view (right). All isodensity values used were 0.05, except for K311 with an isodensity value of 0.042.

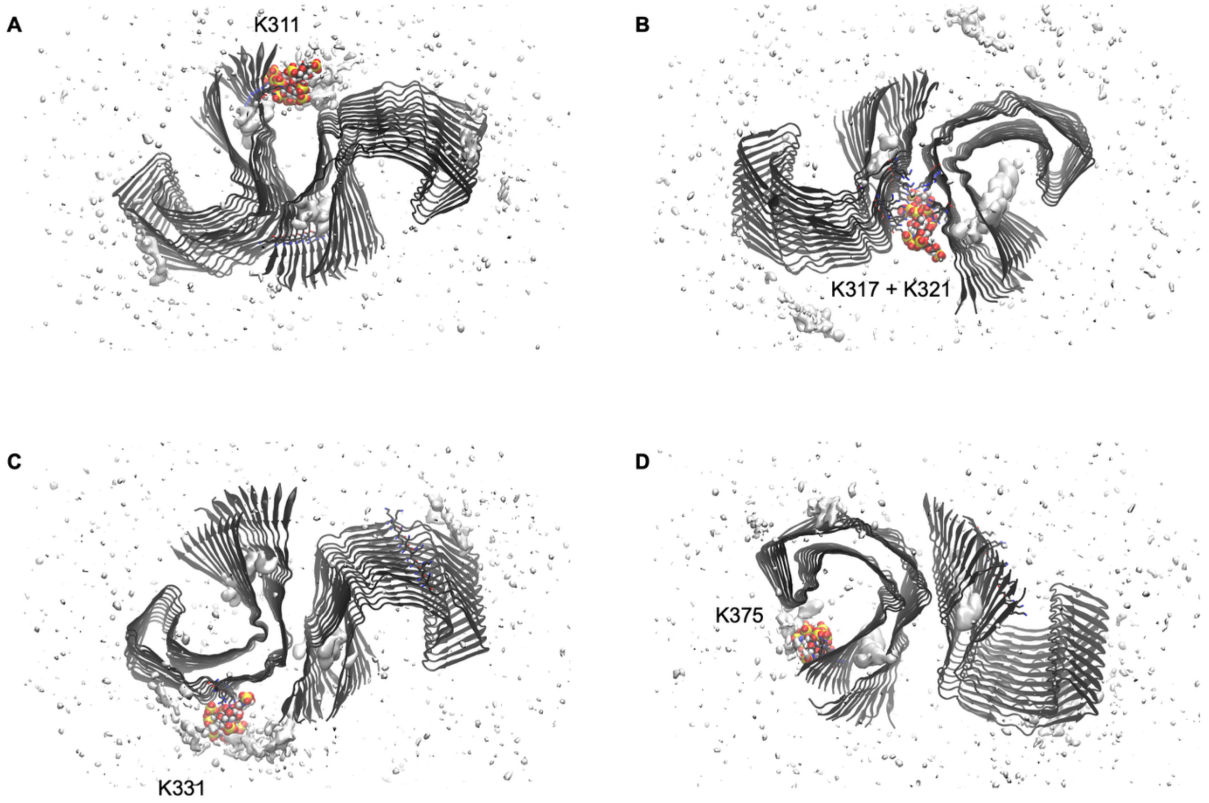


**Figure S8.** Sodium ion density in tau SF simulations. Averaged sodium ion density for one simulation replicate where heparin was bound to (A) K311 (B) K317/K321 (C) K331 and (D) K375. The isodensity value used in all density maps was 0.023.

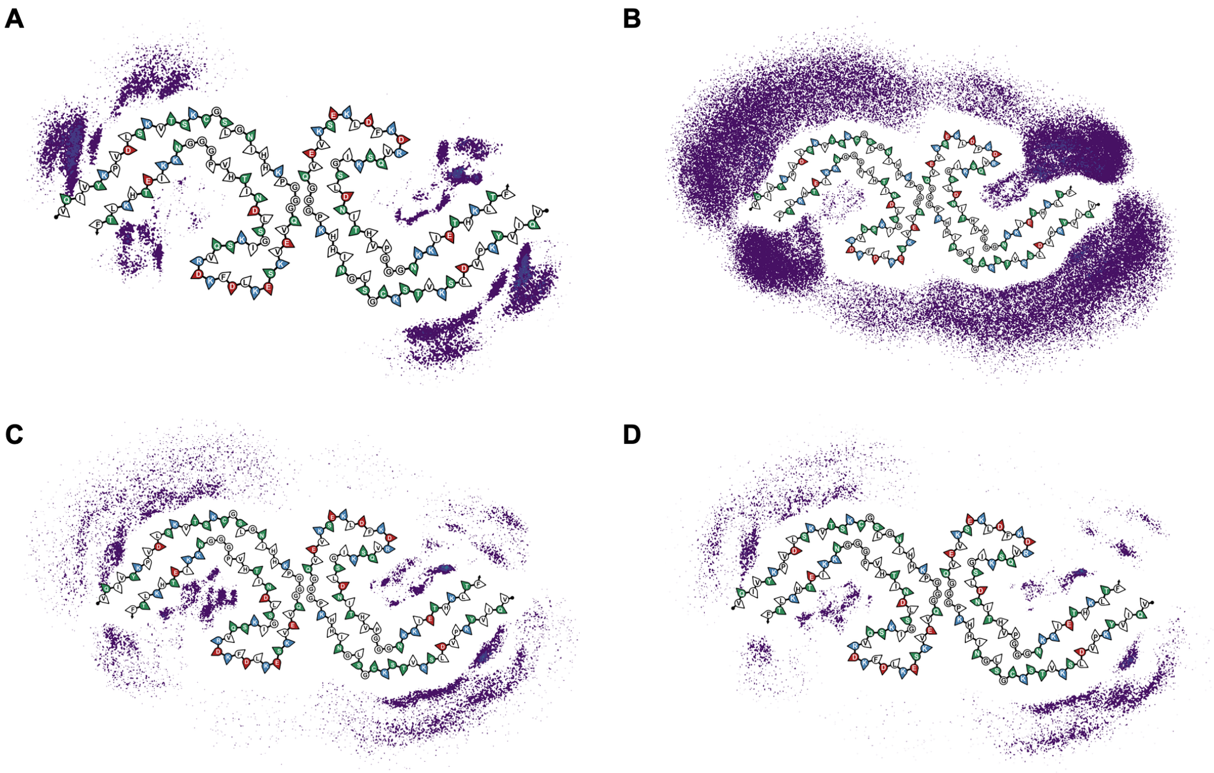


**Figure S9.** Effect of heparin length and sulfation pattern binding modes to PHF fibril core compared to the octasaccharide from Brownian dynamics simulations. The difference in ligand densities for a (A) tetrasaccharide (B) decasaccharide (C) 2-O-desulfated and (D) 6-O-desulfated to the octasaccharide. Positive values of the difference are plotted as the ligand density representing more of a presence of the heparin sulfur atoms in the respective ligand compared to the reference (octasaccharide).

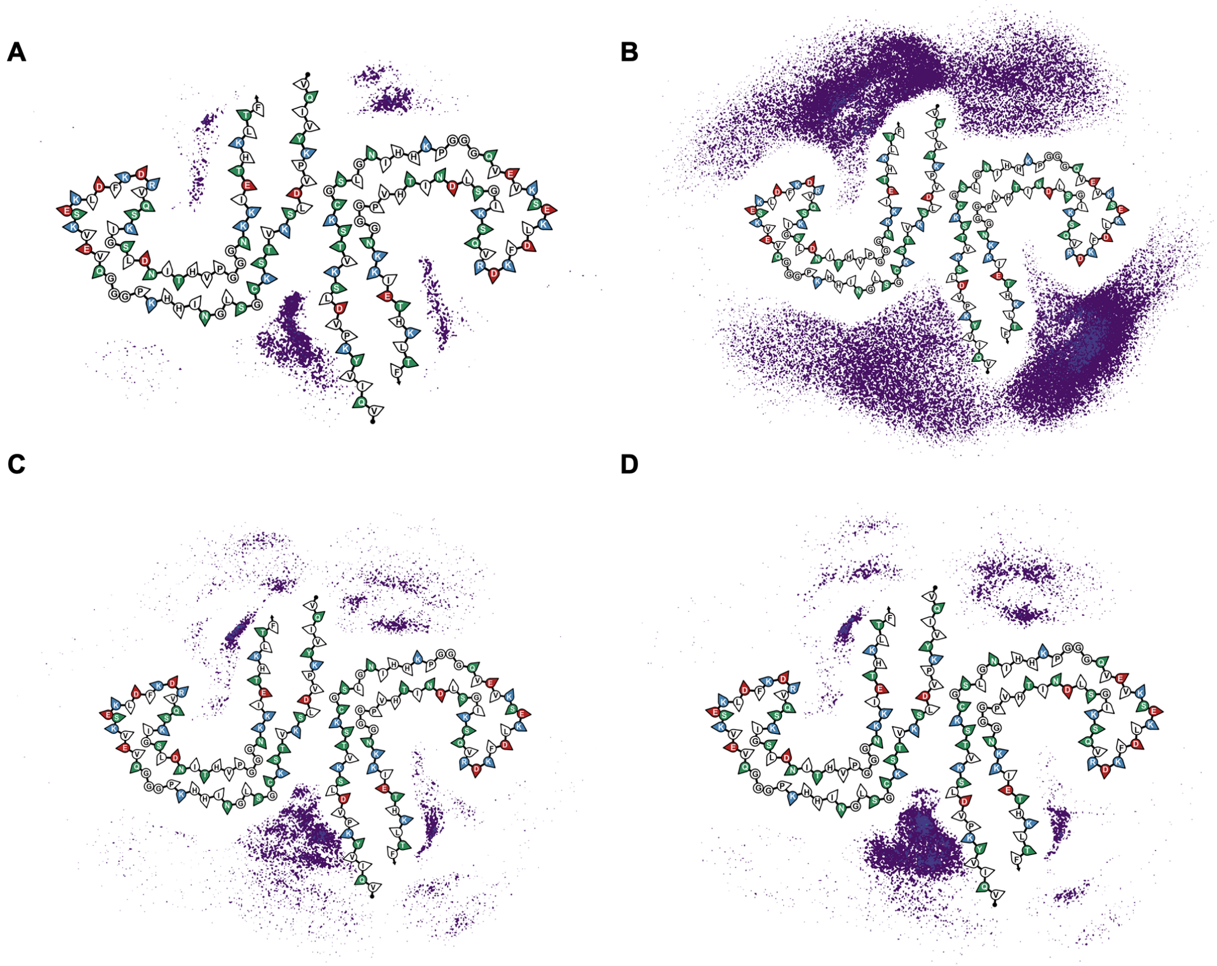


**Figure S10.** Effect of heparin length and sulfation pattern binding modes to SF fibril core compared to the octasaccharide from Brownian dynamics simulations. The difference in ligand densities for a (A) tetrasaccharide (B) decasaccharide (C) 2-O-desulfated and (D) 6-O-desulfated to the octasaccharide. Positive values of the difference are plotted as the ligand density representing more of a presence of the heparin sulfur atoms in the respective ligand compared to the reference (octasaccharide).

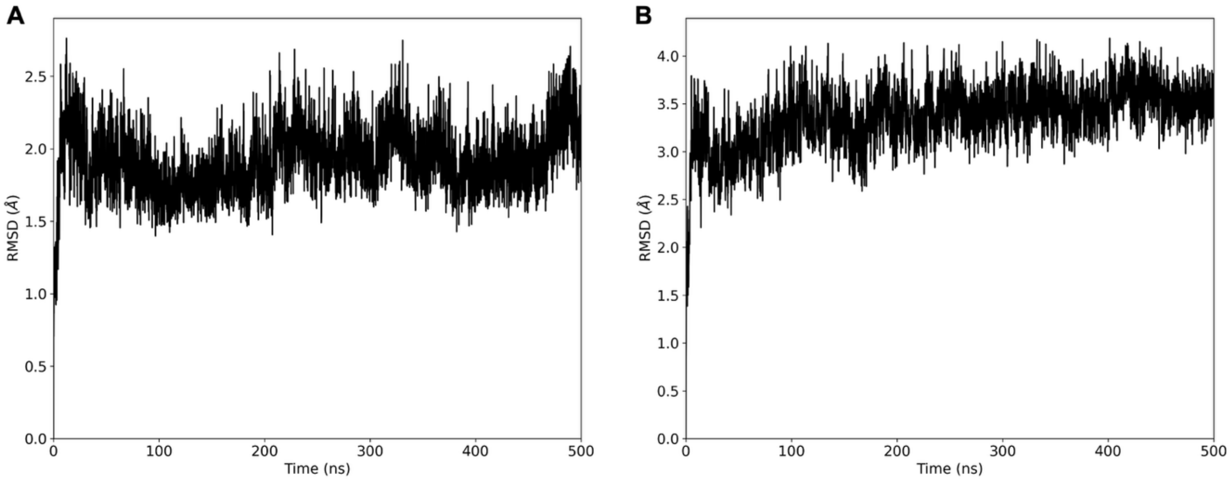


**Figure S11.** RMSD of backbone atoms in simulation of tau fibrils. (A) RMSD of backbone atoms in PHF. (B) RMSD of backbone atoms in SF.

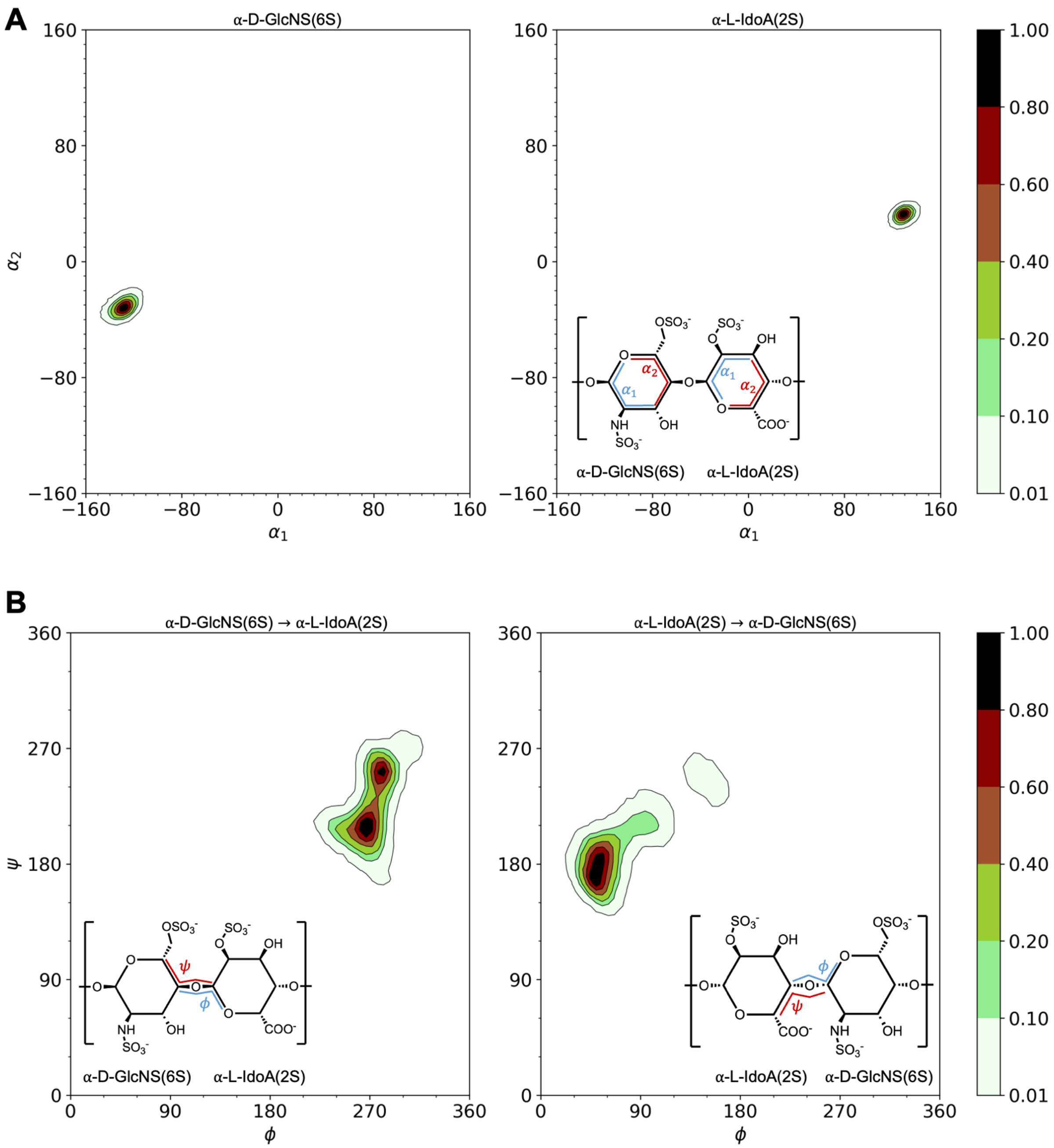


**Figure S12.** Structural characterization of heparin conformations in solution. (A) The relative probability of the dihedral angles $\alpha_1$ (O5-C1-C2-C3) and $\alpha_2$ (C3-C4-C5-O5) corresponding to the preferred ring puckering conformational states for $\alpha$-D-GlcNS(6S) (left) and $\alpha$-L-IdoA(2S) (right). (B) The relative probability of the dihedral angles $\psi$ (C5-C4-O4-C1) and $\phi$ (C4-O4-C1-O5) for the $\alpha$-D-GlcNS(6S) – $\alpha$-L-IdoA(2S) linkage (left) and $\alpha$-L-IdoA(2S) - $\alpha$-D-GlcNS(6S) linkage (right).

**Table S1.** Statistics of the number of unreactive trajectories from Brownian dynamics simulations of the fibril-heparin complexes with varying chain lengths and sulfation patterns of heparin. The number of unreactive trajectories is from a total of 200,000 trajectories from Brownian dynamics simulations. The differing sulfation patterns of heparin were both modified based on a chain length of 8.

| Fibril-Heparin Complex | No. Unreactive Trajectories |
|---|---|
| PHF-8 heparin | 59,187 |
| PHF-10 heparin | 59,434 |
| PHF-4 heparin | 80,314 |
| PHF-2-O-desulfated | 63,677 |
| PHF-6-O-desulfated | 65,779 |
| SF-8 heparin | 71,195 |
| SF-10 heparin | 69,769 |
| SF-4 heparin | 78,164 |
| SF-2-O-desulfated | 77,898 |
| SF-6-O-desulfated | 80,958 |
